# Editorial: Rising stars in urologic oncology: 2021

**DOI:** 10.3389/fruro.2023.1285897

**Published:** 2023-10-10

**Authors:** Juan Gómez Rivas, Riccardo Campi

**Affiliations:** ^1^ Department of Urology, Health Research Institute, Hospital Clinico San Carlos, Madrid, Spain; ^2^ Unit of Urological Robotic Surgery and Renal Transplantation, Careggi Hospital, University of Florence, Florence, Italy; ^3^ Department of Experimental and Clinical Medicine, University of Florence, Florence, Italy

**Keywords:** urology, oncology, prostate, bladder, kidney, cancer, rising stars

In the ever-evolving landscape of medical research and clinical practice, certain individuals emerge as beacons of innovation and promise. In the realm of urologic oncology, a field marked by its dynamic nature and constant advances, the emergence of these rising stars holds immense significance for both patients and the medical community at large. These individuals, driven by their passion, dedication, and groundbreaking contributions, illuminate the path forward for the diagnosis, treatment, and management of urologic cancers.

Urologic oncology encompasses a wide spectrum of conditions, and this Research Topic comprises papers on prostate cancer, bladder cancers, and kidney cancer. The rising stars in this field have shown remarkable prowess in pushing the boundaries of scientific understanding. Their innovative research not only enriches our knowledge base but also serves as a cornerstone for developing more effective therapies. From unraveling the intricacies of cancer biology to harnessing the potential of precision medicine, their work brings us closer to personalized treatments that promise improved patient outcomes.

Investigations in prostate cancer play a pivotal role in unraveling the complexities of this prevalent and clinically diverse disease. The multifaceted nature of prostate cancer demands a comprehensive approach that encompasses various diagnostic tools, biomarkers, imaging modalities, and predictive models. Covering prostate cancer, you will find basic science articles describing new therapies for neurovascular bundles regeneration after radical prostatectomy (Belenchon et al.); describing the protocol for a single-arm pilot feasibility study (IDEAL stage 2a): SAFE (SAlvage Focal irreversible Electroporation)—for recurrent localized prostate cancer (Marra et al.); and on the correlation between a predictive tool called the Immune compleX Predictive Index (iXip) and clinically significant prostate cancer in patients who have undergone radical prostatectomy (https://www.frontiersin.org/research-topics/27264/rising-stars-in-urologic-oncology-2021).

The journey of a rising star in urologic oncology extends beyond the laboratory doors. Their commitment to patient care is nothing short of inspiring. With a deep sense of empathy and a comprehensive approach to treatment, they are shaping how urologic cancers are managed. Through multidisciplinary collaboration, they bridge the gap between research and clinical practice, ensuring that the latest scientific advancements translate directly into enhanced patient care.

On bladder cancer, our investigators found that the platelet-to-lymphocyte ratio values before and during induction therapy could be used as predictors for the progression and recurrence of non-muscle invasive bladder cancer patients receiving bacillus Calmette–Guérin (BCG) immunotherapy (Wu et al.).

One hallmark of rising stars is their remarkable ability to inspire and mentor the next generation of urologic oncologists. Through their guidance, young researchers and clinicians are encouraged to explore uncharted territories, challenge conventional norms, and drive transformative change. The culture of collaboration they foster transcends institutional boundaries and promotes the collective advancement of the field. By nurturing talent and fostering an environment of continuous learning, these rising stars ensure that the torch of innovation remains brightly lit.

Although the ascent of rising stars in urologic oncology is cause for celebration, it is not without its challenges. The journey to transforming scientific discoveries into tangible clinical applications is complex. Funding constraints, ethical considerations, and the need for rigorous validation are among the hurdles that must be navigated. Moreover, the dynamic nature of medical science requires these rising stars to adapt swiftly to emerging technologies and paradigms.

An example of this is the investigations presented on renal cell carcinoma, in which our young investigators found that the expression level of acyl-CoA synthetase long chain family member 3 (*ACSL3*) was significantly reduced in clear cell renal carcinoma tissue, and that its levels of mRNA and protein expression were also significantly lower in both renal cancer cell lines. The level of *ACSL3* could be related in clinics to the clinical stage, progression, and survival (Zhang et al.)

As we gather to recognize and celebrate the achievements of these exceptional individuals, it is evident that the future of urologic oncology is in capable hands. Their unrelenting pursuit of knowledge, a patient-centric approach, and collaborative spirit steer the field toward new horizons. With each innovative study, breakthrough therapy, and empathetic patient interaction, they contribute to a legacy that will shape the trajectory of urologic oncology for years to come.

In addition, in the field of renal cancer, to provide better prognostic information for patients, doctors, and their families, a paper in which a nomogram to predict cancer-specific survival and overall survival in renal cell carcinoma combined with venous tumor thrombus is presented.

In this editorial, we congratulate these rising stars in urologic oncology—trailblazers who illuminate the path toward a future where urologic cancers are better understood, more effectively treated, and, ultimately, conquered.

## Author contributions

JG: Writing – original draft, Writing – review & editing. RC: Writing – review & editing.

